# Permissive human cytomegalovirus infection of a first trimester extravillous cytotrophoblast cell line

**DOI:** 10.1186/1743-422X-1-8

**Published:** 2004-11-17

**Authors:** Heather L LaMarca, Bruno Sainz, Cindy A Morris

**Affiliations:** 1 Interdisciplinary Program in Molecular and Cellular Biology, Tulane University Health Sciences Center, New Orleans, LA, USA; 2 Department of Microbiology and Immunology, Tulane University Health Sciences Center, New Orleans, LA, USA

## Abstract

Human cytomegalovirus (HCMV) is the leading cause of congenital viral infection in the United States and Europe. Despite the significant morbidity associated with prenatal HCMV infection, little is known about how the virus infects the fetus during pregnancy. To date, primary human cytotrophoblasts (CTBs) have been utilized to study placental HCMV infection and replication; however, the minimal mitotic potential of these cells restricts experimentation to a few days, which may be problematic for mechanistic studies of the slow-replicating virus. The aim of this study was to determine whether the human first trimester CTB cell line SGHPL-4 was permissive for HCMV infection and therefore could overcome such limitations. HCMV immediate early (IE) protein expression was detected as early as 3 hours post-infection in SGHPL-4 cells and progressively increased as a function of time. HCMV growth assays revealed the presence of infectious virus in both cell lysates and culture supernatants, indicating that viral replication and the release of progeny virus occurred. Compared to human fibroblasts, viral replication was delayed in CTBs, consistent with previous studies reporting delayed viral kinetics in HCMV-infected primary CTBs. These results indicate that SGHPL-4 cells are fully permissive for the complete HCMV replicative cycle. Our findings suggest that these cells may serve as useful tools for future mechanistic studies of HCMV pathogenesis during early pregnancy.

## Findings

Human cytomegalovirus (HCMV) is a ubiquitous beta-herpesvirus that is the leading cause of congenital viral infection in the United States and Europe. Intrauterine transmission of the virus occurs in approximately 40% of pregnant women with primary HCMV infection, and the incidence of congenital HCMV infection is an estimated 1% of newborns [1-3]. Although the pathogenesis of HCMV transmission to the fetus during pregnancy is unclear, the placenta has been implicated as an important determining factor [4-8]. Primary first trimester extravillous cytotrophoblasts (CTBs), which are specialized placental epithelial cells that invade and remodel the uterine wall during placentation, have been previously shown to be fully permissive for HCMV infection *in vitro *[7,9]. Additionally, using an *in vitro *coculture system, Maidji and colleagues demonstrated that infected uterine microvascular endothelial cells transmit HCMV to differentiating invading CTBs, suggesting that placental HCMV infection can occur in a retrograde fashion that initiates in the maternal endothelium [8]. Despite these reports, the minimal mitotic potential of primary CTBs restricts experimentation to a few days, which may be problematic for mechanistic studies of the slow-replicating virus. Alternatively, the utilization of trophoblast cell lines would provide an easily manipulative *in vitro *model for the study of HCMV infection of the placenta. In the present study, we used a first trimester human extravillous CTB cell line, termed SGHPL-4, to investigate HCMV replication. SGHPL-4 cells were derived from first trimester chorionic villous tissue and have been described previously. Importantly, these cells share many characteristics with isolated primary cells, including the expression of cytokeratin-7, HLA class I antigen, HLA-G, BC-1, CD9, human chorionic gonadotrophin, and human placental lactogen[10-12].

**Figure 1:**
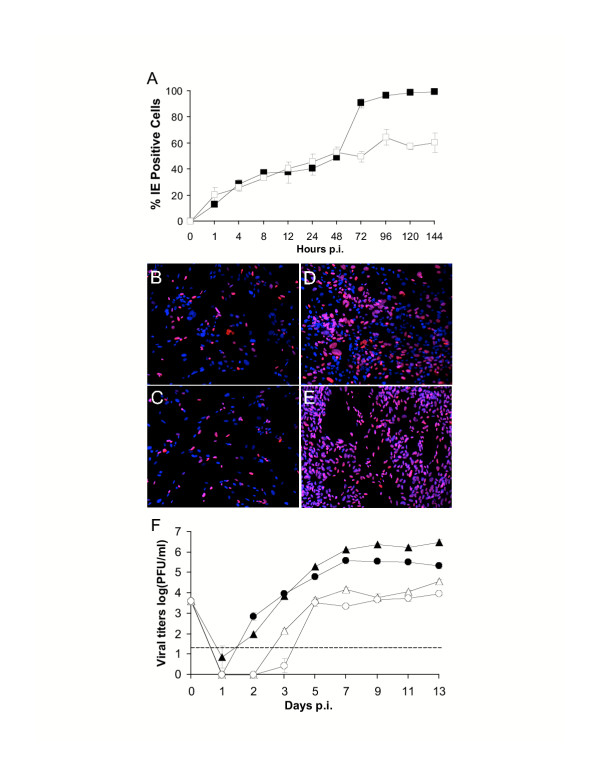
**Productive HCMV infection in SGHPL-4 and HFF cells. **(A-E) HCMV IE protein expression in human cytotrophoblasts. SGHPL-4 (□) or HFF (■) cells were infected with HCMV strain RVdlMwt-GFP [17] at a MOI of 2.5 PFU per cell and incubated at 37°C for 1, 4, 8, 12, 24, 48, 72, 96,120 or 144 h. At the indicated times, cells were fixed and stained for HCMV IE 1/2 and DAPI (Molecular Probes) and visualized on a Zeiss Axio Plan II microscope (Thornwood, NY). To determine the number of HCMV-infected cells, three fields of view were considered and the percent of IE-positive cells was calculated as: (average number of IE-stained cells/average number of DAPI-stained cells) × 100. The graph demonstrates an increase in the percentage of SGHPL-4 and HFF cells expressing IE 1/2 over a period of time. Representative images of HCMV IE 1/2 are depicted at 8 h p.i in (B) CTBs and (C) HFFs and at 120 h p.i. in (D) CTBs and (E) HFFs; IE 1/2-red, DAPI-blue, overlaid-purple. (F) Infected CTBs produce and release infectious virions. SGHPL-4 or HFF cells were inoculated with HCMV at a MOI of 0.1 PFU per cell. At the indicated times, cells or culture medium were harvested, freeze-thawed three times, and titers of infectious virus in SGHPL-4 cell lysates (○) and supernatants (△) and HFF cell lysates (●) and supernatants (▲) were determined by a microtiter plaque assay on HFFs [18]. Infectious progeny virus was detected in both cell lysates and culture supernatants of SGHPL-4 and HFF cells. The dashed line represents the lower limit of detection of the plaque assay used to measure viral titers.

The lytic replication cycle of HCMV is a temporally regulated cascade of events that is initiated when the virus binds to host cell receptors. Upon entry into the cell, the viral DNA translocates to the nucleus where viral gene expression occurs in a stepwise fashion beginning with the expression of immediate early (IE) genes (reviewed in [13]). To initiate studies of HCMV infection in the SGHPL-4 cell line, placental CTBs and human foreskin fibroblasts (HFFs) were infected with HCMV and the nuclear HCMV IE proteins (IE 1/2; Chemicon, Temecula, CA) were examined by immunofluorescence at various intervals after viral infection. At 3 h p.i., IE 1/2 was present in SGHPL-4 cells in similar numbers to that of HFFs. In fact, the percentages of IE-positive cells initially did not differ between CTBs and HFFs, suggesting that viral entry into the cells and IE transcription occurred at similar rates between the cell types (Figure 1A,1B,1C). Characteristic cytopathic effects of HCMV infection including swollen cells with nuclear inclusions were observed in both SGHPL-4 and HFF cells by 48 h p.i. (data not shown), and throughout a 6 day culture period, the numbers of IE-positive cells increased continuously in both cell types (Figure 1A). Interestingly, the rate of IE 1/2 protein expression in SGHPL-4 cells as compared to HFFs appeared to differ beginning at 72 h p.i. By 72 h p.i., there was a 40% increase in the percentage of IE-positive HFFs over SGHPL-4 cells. While nearly 100% of HFFs stained positive for IE 1/2 120 h (5 days) p.i., the maximum fraction of IE-positive SGHPL-4 cells did not exceed 60% (Figure 1A,1D,1E), suggesting that subsequent viral gene expression and thus cell-to-cell viral spread may be kinetically delayed. These findings are consistent with other reports demonstrating delayed kinetics of viral gene expression in primary CTBs as compared to primary fibroblasts [14].

Although several studies have shown that first trimester primary trophoblasts can be permissively infected with HCMV, some reports have demonstrated that progression through the replicative cycle was slow and progeny virus remained predominantly cell associated [9,15,16]. To determine whether SGHPL-4 cells support productive HCMV replication, 9 day viral growth assays were performed (Figure 1F). SGHPL-4 and HFF cells were inoculated with HCMV at a MOI of 0.1 PFU per cell, and both culture lysates and supernatants were titered for infectious virus at various days p.i. While viral titers in infected HFFs were detectable as early as 2 days p.i., viral replication was undetectable or below the lower limit of detection of the assay in SGHPL-4 lysates up to 3 days p.i. However, at days 5–9 p.i., HCMV replicated to titers of ≥ 5000 and 3600 PFU/ml in SGHPL-4 cell lysates and supernatants, respectively. Relative to HFF-infected control cultures, viral titers recovered from SGHPL-4 culture lysates and supernatants were reduced by ~20- and ~200-fold, respectively (Figure 1F). While viral titers were decreased in infected SGHPL-4 cells as compared to infected HFFs, placental CTBs effectively supported productive viral replication as measured by infectious intracellular and extracellular virions. Moreover, when SGHPL-4 cells were infected with another laboratory-derived strain of HCMV (strain AD169), similar results were obtained (data not shown) suggesting that viral replication was not virus-strain specific. Collectively, these data indicate that SGHPL-4 cells support productive HCMV replication.

In the present study, we demonstrate that the first trimester extravillous CTB cell line SGHPL-4 is fully permissive for HCMV replication. The utilization of a CTB cell line, rather than primary CTBs and explant cultures that are short-lived cultures, may provide an experimental advantage for *in vitro *studies of placental HCMV infection. We propose that the permissiveness for HCMV replication in SGHPL-4 cells may allow for future studies in elucidating the molecular mechanism(s) of HCMV infection and pathogenesis at the maternal-fetal interface during early pregnancy.

## List of abbreviations

human cytomegalovirus (HCMV), cytotrophoblast (CTB), human foreskin fibroblasts (HFFs), immediate early (IE), hours (h), post-infection (p.i.), multiplicity of infection (MOI), plaque forming unit (PFU), 4', 6-diamidino-2-phenylindole, dihydrochloride(DAPI)

## Competing interests

The authors declare that they have no competing interests.

## Authors' contributions

HL participated in the experimental design, performed all experiments and drafted the manuscript. BS participated in the experimental design and assisted with viral propagation and viral replication assays. CM conceived of the study and participated in its design and coordination. All authors read and approved the final manuscript.
